# The L2 decomposition of transparent derived verbs - Is it ‘morphological’? A commentary on De Grauwe, Lemhöfer, Willems, & Schriefers (2014)

**DOI:** 10.3389/fnhum.2015.00220

**Published:** 2015-04-23

**Authors:** Gunnar Jacob

**Affiliations:** Potsdam Research Institute for Multilingualism, University of PotsdamPotsdam, Germany

**Keywords:** morphological processing, derivational affixes, decomposition, non-native speakers

Assume you come across a morphologically-complex Japanese word such as “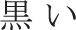.” Even if you have absolutely no knowledge of Japanese at all, and are therefore completely insensitive to the word's morphological structure, you might still be able to distinguish between the stem “
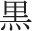
” and the affix “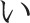.” This is because in Japanese, stems are typically written in Kanji, while affixes are written in Hiragana, with the surface form differences between these two scripts being distinct enough that they might even be noticeable for someone without any knowledge of Japanese. As a result, you might actually be able to “decompose” the word, but this decomposition process obviously does not operate on morphological units. Instead, you simply make use of the fact that, in addition to being *morphological* units, the head “
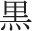
” and the affix “
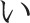
” also constitute units on a completely different level; they are at the same time also *orthographic* units.

How is this (admittedly rather far-fetched) example related to De Grauwe et al. (2014) study on morphological decomposition in non-native (L2) speakers? In their fMRI experiment, De Grauwe and colleagues convincingly show that L2 speakers of Dutch, just as native speakers, are able to decompose transparent derived verbs such as “*opstaan*” into the head “*staan*” and the modifier “*op*.” Based on these findings, the authors argue against accounts of L2 morphological processing which assume qualitative differences between native speakers and L2 speakers with regard to morphological decomposition.

In De Grauwe's study, stems and affixes were of course not written in different scripts. However, just as in the Japanese example, the head “*staan*” and the modifier “*op*” in a Dutch word such as “*opstaan*” are not only morphological units, but also constitute units on other linguistic levels. First, at least for the vast majority of the materials used in De Grauwe's study, head and modifier are also existing *lexical* units. Specifically, “*op*” is a Dutch preposition, while “*staan*” is a verb. For separable verbs (which constitute 55 out of 70 verbs used in the experiment), this is actually the case by default, assuming that such verbs are either “phrasal constructs” (Booij, 1990) or derived through incorporation of a preposition into a verb (Van Riemsdijk, 1978). As a result, modifiers in separable verbs automatically also have to be existing words of their own. Second, “*op*” and “*staan*” also constitute *syntactic* units (Booij, 2002). While verbs are usually syntactic islands (i.e., a syntactic operation such as inflection is normally conducted on the entire verb), separable verbs are an exception to this; for example, in order to produce a grammatically correct Dutch sentence based on the verb “*opstaan*,” such as “*Marie staat op*,” the formulator has to separate head and modifier, and subsequently perform different syntactic operations (e.g., inflecting the head, moving each unit to its correct position in the sentence) on each of the two.

Thus, while the effects reported in De Grauwe's study presumably involve a form of decomposition, the particular properties of the derived verbs used in the study raise the question whether this decomposition mechanism really operates on morphological units. In other words, even a parser which is completely insensitive to morphology might be able to decompose “*opstaan*” into “*op*” and “*staan*,” provided that it has access to either information about syntactic properties of separable verbs or to a lexicon which contains separate entries for “*op*” and “*staan*.”

A possible counter-argument against this is based on the particular area for which the decomposition effect occurred in the fMRI study. De Grauwe and colleagues correctly point out that the effect occurred in the LIFG, an area which, in several previous papers, has been found to play a role in morphological decomposition. However, it could simply be that the LIFG is generally involved in all sorts of decomposition processes. The same knife can theoretically be used to cut all sorts of different things into pieces.

Given these particular linguistic properties of their materials, how does De Grauwe's study relate to the current debate about L1/L2 differences in morphological processing? While previous behavioral studies investigating the L2 processing of derived forms (e.g., Silva and Clahsen, 2008; Clahsen and Neubauer, 2010; Diependaele et al., 2011; Kirkici and Clahsen, 2013) have come to different conclusions about L2 processing, all of these studies have discussed their findings with reference to the early morpho-orthographic segmentation mechanism proposed by Rastle et al. (2004) and Marslen-Wilson (2007). Crucially, this account assumes a decomposition mechanism which operates specifically on *morphological* units (in Rastle's case, morphemes; in Marslen-Wilson's case, affixes). Unlike De Grauwe and colleagues, the L2 studies mentioned above used derived forms in which stems and affixes constitute units only at the morphological level, and also, through appropriate control conditions, went to great lengths to ensure that priming effects are morphological in nature. Thus, while De Grauwe and colleagues interpret their findings as evidence against L1/L2 differences, the linguistic properties of their materials make it difficult to discuss their findings with reference to these previous studies. In this respect, behavioral studies which have found similar priming effects for derived forms in L1 and L2 speakers (e.g., Diependaele et al., 2011) can possibly be considered more direct evidence against the idea of fundamental differences between L1 and L2 processing. Additionally, de Grauwe's study also differs from these previous studies with regard to the methodological approach (long-lag priming vs. masked priming) and with regard to the possible role of the L1 in L2 processing (all stimuli were Dutch/German cognates), making the studies difficult to compare.

Importantly, these issues do not diminish De Grauwe's contribution to the field in any way. Their fMRI study quite convincingly shows that L2 speakers do not have a general problem with the decomposition mechanism *per se*. Also, De Grauwe's claims about the processing of the particular class of verbs investigated in the study, and the lack of fundamental L1-L2 differences with regard to these verbs, remain valid. The key question is whether these findings can be generalized from this particular verb class to all derivations, or whether such verbs possess specific linguistic properties which make them uniquely different from other types of morphologically-complex forms. Hence, it would be interesting to see whether L2 speakers show similar effects for types of morphologically-complex words in which stems and affixes only constitute units at the morphological level, such as derived nominalizations or even inflected forms.

## Conflict of interest statement

The author declares that the research was conducted in the absence of any commercial or financial relationships that could be construed as a potential conflict of interest.
